# Prostate cancer detection using e-nose and AI for high probability assessment

**DOI:** 10.1186/s12911-023-02312-2

**Published:** 2023-10-06

**Authors:** J. B. Talens, J. Pelegri-Sebastia, T. Sogorb, J. L. Ruiz

**Affiliations:** 1 Sensor and Magnetism Group, Institut de Recerca Per a La Gestió Integrada de Zones Costaneres (IGIC), Campus de Gandia, Universitat Politecnica de Valencia, Paranimf 1, Grao de Gandia, 46000 Valencia, Spain; 2 Educacion, Conselleria de Educacion, Cultura y Deporte, Av. de Campanar, 32, 46015 Valencia, Spain; 3 Surgery Department, Universitat de Valencia, Av Fernando Abril, Martorell, 106., 46026 Valencia, Spain

**Keywords:** Deep learning, Neural networks, Machine intelligence, e-Nose, MOOSY-32, Prostate cancer

## Abstract

This research aims to develop a diagnostic tool that can quickly and accurately detect prostate cancer using electronic nose technology and a neural network trained on a dataset of urine samples from patients diagnosed with both prostate cancer and benign prostatic hyperplasia, which incorporates a unique data redundancy method. By analyzing signals from these samples, we were able to significantly reduce the number of unnecessary biopsies and improve the classification method, resulting in a recall rate of 91% for detecting prostate cancer. The goal is to make this technology widely available for use in primary care centers, to allow for rapid and non-invasive diagnoses.

## Introduction

**Fig. 1:**
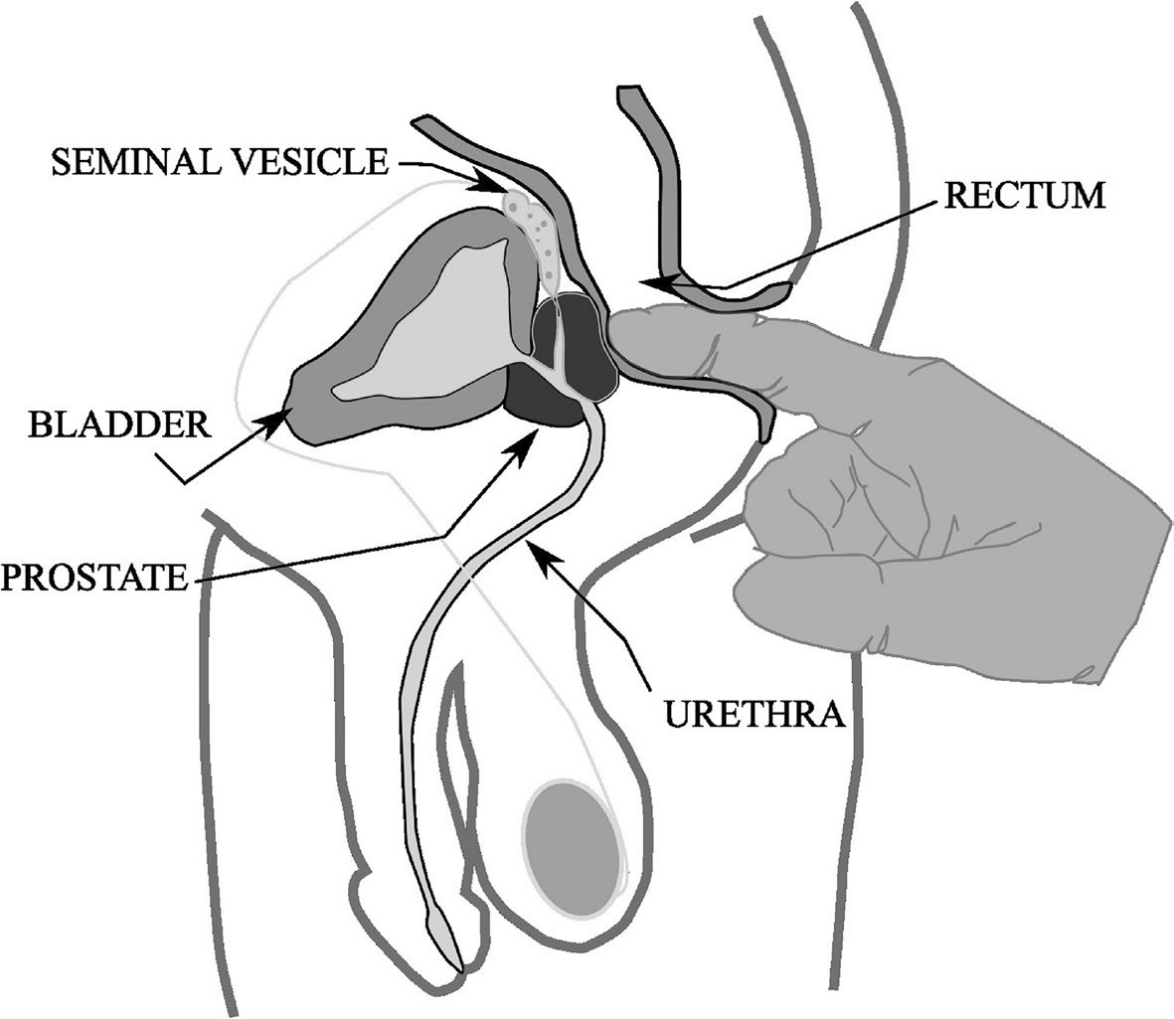
Invasive rectal tract rendering

Prostate cancer is one of the most common types of cancer in men, and early detection is critical for effective treatment. However, current methods for detecting prostate cancer, such as biopsies and digital rectal examination, are invasive and can lead to a high number of unnecessary procedures. The most commonly used biological marker for prostate cancer detection is PSA, or Prostate-Specific Antigen, but it is not specific to cancer and can lead to false positives in some situations such as benign prostatic hyperplasia, normal ejaculation, urinary retention [1], infection, or some gland inflammation [2]. Therefore, there is a need for a non-invasive method for detecting prostate cancer [3], as ilustrated in Fig. 1.

Over the last thirty years metal oxide semiconductor technology (MOS) applied to the detection of substances has been effective for many fields of science and industry: spirits [4] and [5], toxic gases [6], tobacco [7] or smoke [8], and medical applications [9]. The electronic nose (e-Nose), like the human being, has two major branches, acquisition, and processing. Chemical sensors react to certain substances and these reactions are processed through artificial intelligence software [10].

However, what sets our work apart is the pioneering approach we introduce. By harnessing a dataset of urine samples from patients diagnosed with both prostate cancer and benign prostatic hyperplasia, we have achieved a groundbreaking 91% recall rate for prostate cancer detection. This remarkable breakthrough, coupled with our novel neural network design and data redundancy method, not only reduces unnecessary biopsies but also positions the electronic nose as a transformative tool for primary point-of-care applications in the near future [11].

In recent decades, early and accurate cancer detection has become a crucial objective for improving survival rates and the quality of life for patients. The combination of cutting-edge technology, artificial intelligence, and data analysis has revolutionized the field of oncology, opening new avenues for early detection and characterization of different types of cancer. In this context, our research is situated at the intersection of these disciplines with the goal of developing an innovative diagnostic tool that harnesses electronic nose technology and artificial intelligence for non-invasive prostate cancer detection.

To fully appreciate the relevance of our work, it is essential to recognize the significant contributions of prior research that have propelled the field of cancer detection and phenotyping. Among the most influential studies are the following:

D'Orazio et al [12] this pioneering study addressed the understanding of cancer cell behavior through motility and shape characteristics. It introduced techniques such as peer prediction and dynamic selection, which have been instrumental in enhancing cancer diagnosis and treatment.

D’Orazio et al [13] this study marked a significant advancement by applying machine learning techniques and time-lapse microscopy to monitor gene expression and drug responses in colorectal adenocarcinoma cells. Its focus on phenomics has influenced the study of genetic and phenotypic variability in cancer.

Mencattini et al [14] this work has demonstrated the importance of optimal feature selection in real-time cell imaging analysis. The "Deep-Manager" tool developed in this study has provided a solid foundation for optimizing feature extraction in cellular imaging analysis, which is relevant to cancer phenotyping.

In summary, while these previous investigations may not be directly related to our work, they have established a robust framework for it and illustrate the ongoing evolution in cancer detection and characterization. Our research contributes to this body of knowledge by developing an innovative diagnostic tool that leverages electronic nose technology and artificial intelligence, with the potential to revolutionize early prostate cancer detection and reduce the need for invasive procedures.

## Methods and procedures

In order to conduct a thorough machine learning study, a dataset must be created. The size of this dataset is crucial for the research, and in this case, it was compiled from patients with various stages of prostate cancer (CaP) and benign prostatic hyperplasia (HBP). As a result, the dataset includes two distinct patient groups: those with CaP and those with HBP. The study was approved by the Ethical Committee on Clinical Research of the Hospital Universitari i Politècnic La Fe de Valencia (Spain) in compliance with the Declaration of Helsinki. Registration number ethics of CEIC: 2022-191-1 with date 30/03/2017.

### Getting dataset

In this study, we employed the MOOSY-32 electronic nose [15] to acquire voltage response curves from metal oxide semiconductor sensors when exposed to urine gas from patients. The device is equipped with four different types of Figaro sensors, arranged in thirty-two sockets. Tables 1, 2, 3 and 4 present the reference and sensitivity of the sensors to various substances. The choice of these sensors was based on their compatibility with the MOOSY-32 and the availability of Figaro datasheets displaying their sensitivity characteristics. Equation 1 illustrates the correlation between gas concentration in parts per million (*C*) and the resistivity obtained (*R*_*s*_).

**Table 1:** Data *A* and *α* from Figaro Sensor TGS-2611E00 [16]

Gas	*A*	*α*
Methane	29.37996766	-0.3948275158
Isobutane	8.715280172	-0.009612300255
Hydrogen	26.35859083	-0.3145073661
Propane	-	-
Ethanol	8.592741295	0.0
Air	8.584894051	0.0
CO	-	-

**Table 2:** Data *A* and *α* from Figaro Sensor TGS-2611C00 [17]

Gas	*A*	*α*
Methane	38.98893094	-0.4294309742
Isobutane	48.40386618	-0.3910578991
Hydrogen	41.48221372	-0.3609195915
Propane	-	-
Etalon	55.17922683	-0.3750828308
Air	8.584894051	0.0
CO	-	-

**Table 3:** Data *A* and *α* from Figaro Sensor TGS-2610C00 [18]

Gas	*A*	*α*
Methane	62.22614045	-0.5290784226
Isobutane	76.61767488	-0.58687488
Hydrogen	83.58818144	-0.5147845058
Propane	79.41376581	-0.52781418
Ethanol	127.3119505	-0.5258350324
Air	10.54330923	-0.001766784452
CO	-	-

**Table 4:** Data *A* and *α* from Figaro Sensor TGS-2620 [19]

Gas	*A*	*α*
Methane	79.49594193	-0.4602952512
Isobutane	26.88377546	-0.5979539273
Hydrogen	17.6124846	-0.5785034943
Propane	-	-
Ethanol	35.89744669	-0.7083796675
Air	17.58233645	0.0
CO	47.99739024	-0.6086910204

1 $${R}_{s}=A{[C]}^{a}$$

By utilizing graphical representation and the regression analysis tool in spread- sheets, we were able to determine the values of *A* and *α* for each sensor. The results are presented in Tables 1, 2, 3 and 4.

With the ability to accurately approximate the parts per million (ppm) value using the gas sensor, we incorporated an additional seven parameters into our dataset for further analysis.

To increase the size of our dataset, we employed a strategy of redundancy. Using five milliliters of urine per container and collecting four containers per patient, we obtained samples from forty patients. By utilizing the MOOSY-32 electronic nose, we acquired five sets of data from each container, resulting in a total of 800 files, each containing 32 curves.

**Fig. 2:**
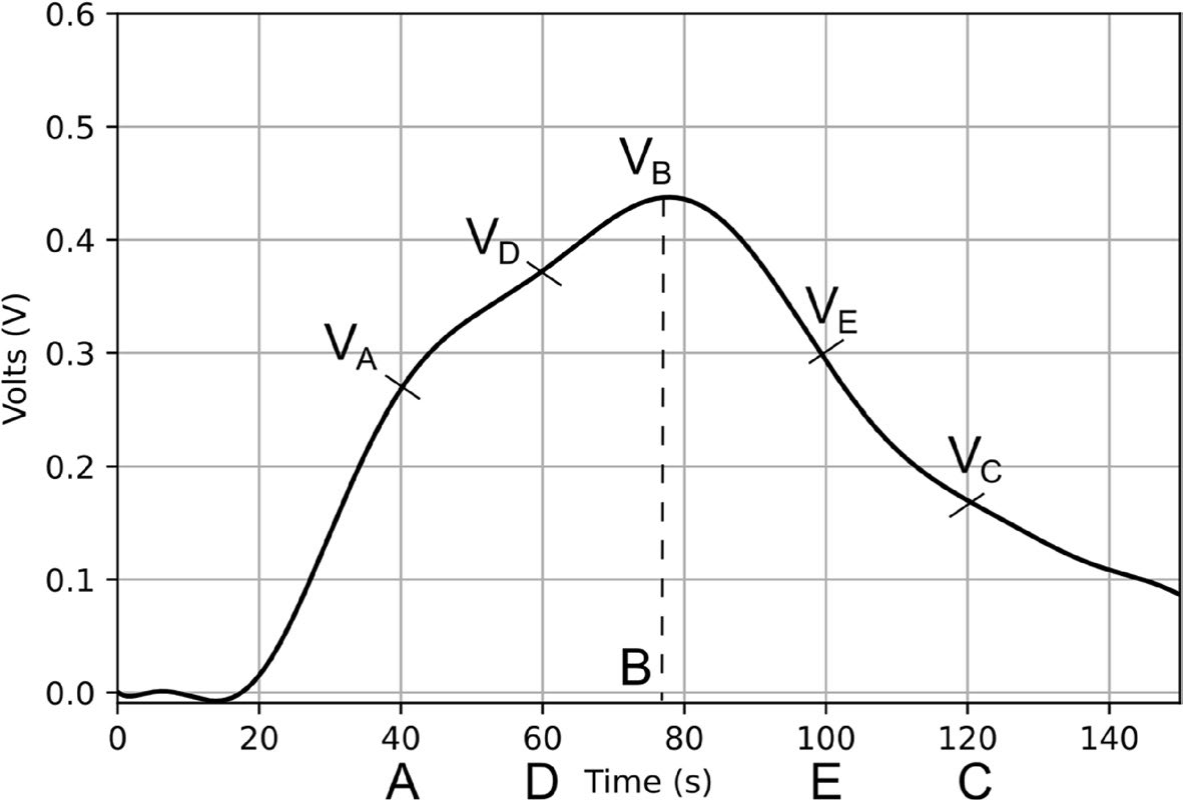
Setting points at curves

The foundation of our data is the curve, which is essentially an array of voltage values with a size of 15000 points. After implementing filters and addressing the offset, the curve appears as depicted in Fig. 2. By extracting data from specific points on the curve, such as *V A* (*t* = 40*s*), *V D* (*t* = 60*s*), *V B* = *V*_max_, *V E* (*t* = 100*s*), and *V C* (*t* = 120*s*), we are able to calculate other parameters, as outlined in Eqs. 2-12.

2 $${\mathrm{slope}}_{AB}=\frac{VB-VA}{tB-tA}$$

3 $${\mathrm{slope}}_{BC}=\frac{VC-VB}{tC-tB}$$

4 $${\mathrm{slope}}_{AD}=\frac{VD-VA}{tD-tA}$$

5 $${\mathrm{slope}}_{DE}=\frac{VE-VD}{tE-tD}$$

6 $${\mathrm{slope}}_{EC}=\frac{VC-VE}{tC-tE}$$

7 $${\mathrm{slope}}_{BE}=\frac{VE-VB}{tE-tB}$$

8 $${\mathrm{slope}}_{DB}=\frac{VB-VD}{tB-tD}$$

9 $${\mathrm{dif}}_{BA}=VB-VA$$

10 $${\mathrm{dif}}_{BC}=VB-VC$$

11 $${\mathrm{dif}}_{BD}=VB-VD$$

12 $${\mathrm{dif}}_{BE}=VB-VE$$

To achieve 32 parameters in our dataset, we incorporated eight statistical data points: 75th percentile, standard deviation, mode, mean, median, interquartile range, coefficient of variation, asymmetry coefficient, and a unique identifier created using the sensor and socket name. This resulted in a total of 640 instances for each patient, each with 32 parameters.

### Neural network

**Fig. 3:**
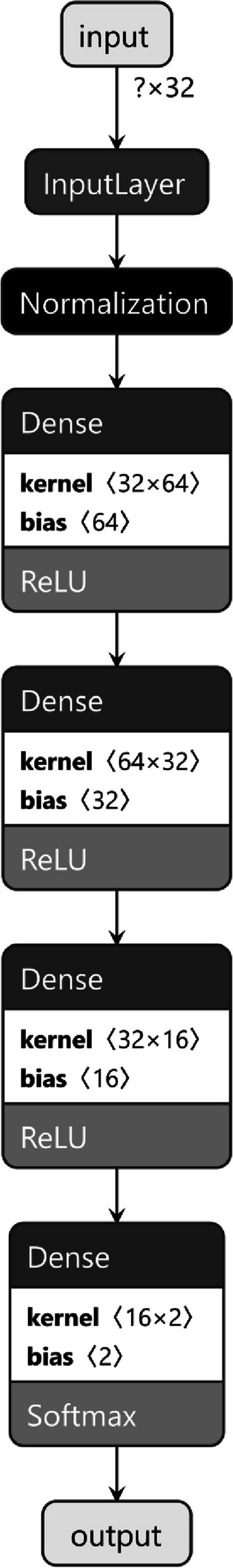
Neural network model from Netron App representation

To construct the neural network, we utilized the Python libraries Tensorflow and Keras [20]. The network architecture is illustrated in Fig. 3. The first layer consists of an input layer with 32 neurons, corresponding to the number of input parameters. The second layer is a normalization layer. The third layer includes a hidden layer with 64 neurons and a ReLU [21] activation function. Two additional hidden layers were also incorporated, with a reduction in the number of neurons from 64 to 16. The final layer has two neurons and utilizes a SoftMax activation function, which produces the probability of the sample belonging to each class. To compile the network, we set the bias initializer to ’zeros’ and the kernel to ’glorot uniform’ . The optimizer used is the SGD optimizer with a learning rate of ’0.001’, decay of ’1e-7’, momentum of ’0.9’, loss function of ’categorical crossentropy’, and metrics of ’accuracy’.

### Training

To train the neural network, we divided the dataset into two groups: a training set and a test set, each containing instances from 20 patients. The training set includes instances from 10 patients with CaP and 10 patients with HBP, resulting in a total of 12,800 instances for training. The test set includes the same number of instances, but from different patients. Before commencing the training process, we set the batch size to 32 and the number of epochs to 1,280.

## Results

**Fig. 4:**
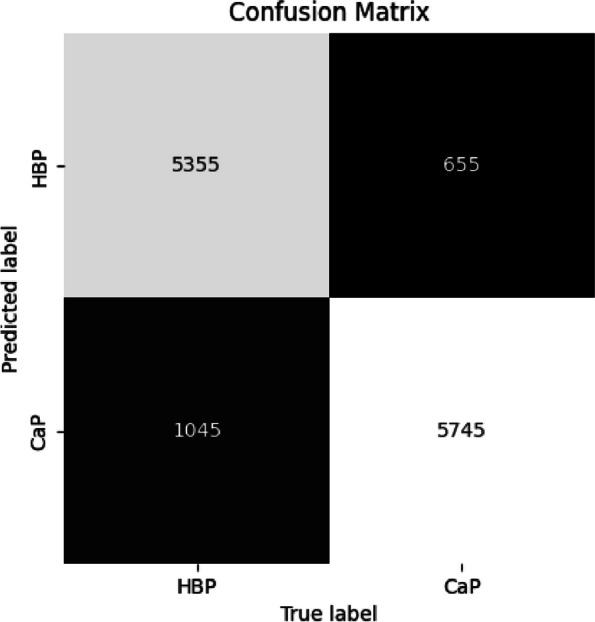
Confusion matrix from test-set

The confusion matrix, generated by evaluating the test set using the neural network, is illustrated in Fig. 4. The training set and test set were labeled as 0 for HBP and 1 for CaP, resulting in the normal representation of the confusion matrix where the false negative is in the first quadrant and the false positive in the third.

**Fig. 5:**
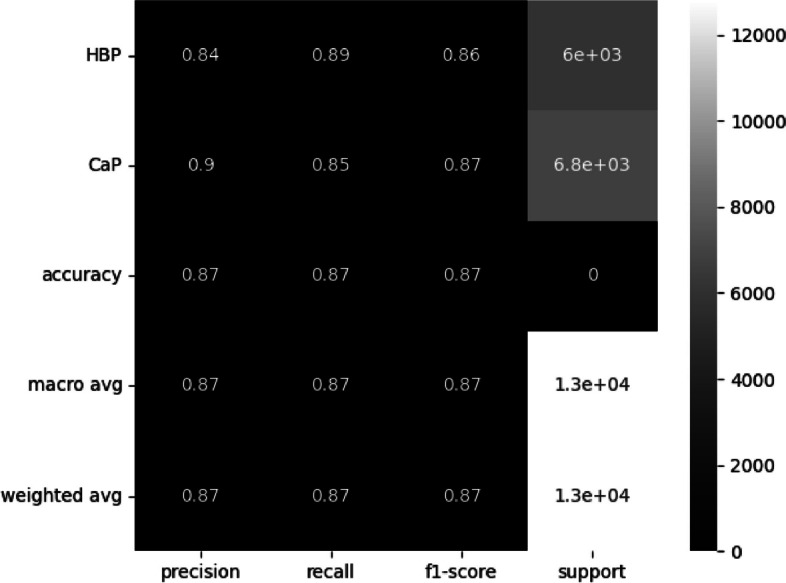
Classify statistics results

The number of instances from patients with cancer that were classified as HBP is high and surpasses that of a single patient. Despite this, as shown in Fig. 5, the accuracy is 87%. However, for clinical sense, it is necessary to improve the recall for HBP.

**Fig. 6:**
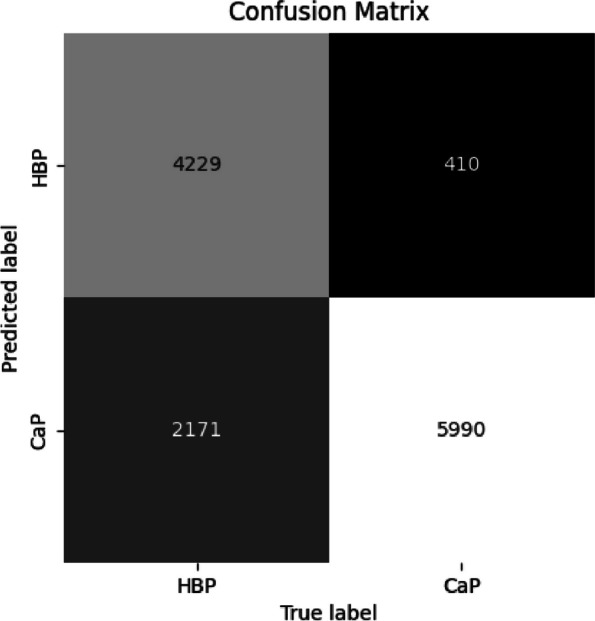
Confusion matrix from test-set

**Fig. 7:**
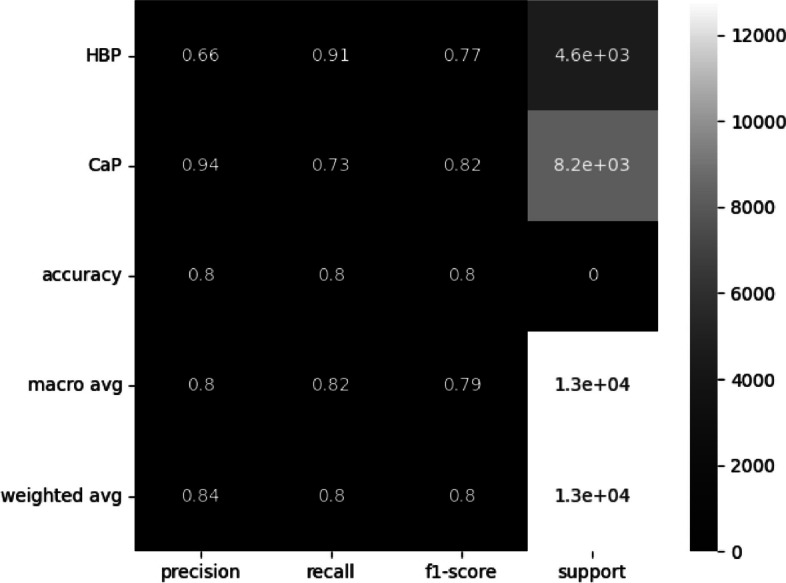
Classify statistics results

In order to enhance the recall, the class weight was set to 0:1.0, 1:32.0 with 0 representing HBP and 1 representing CaP, and the neural network was retrained. The results are illustrated in Figs. 6 and 7.

## Discussion

Our research has culminated in the development of an effective neural network for prostate cancer detection, utilizing MOOSY-32 electronic nose technology and artificial intelligence techniques. In this section, we will discuss the results and their significance, along with the implications and limitations of our study.

### Interpretation of results

The findings of our study indicate that the neural network we developed is highly accurate in classifying cases of prostate cancer. The high level of precision, with a recall rate of 91%, suggests that our methodology has the potential to significantly reduce the need for invasive biopsies and enhance early detection of this disease.

### Clinical implications

These results hold significant clinical implications. Reducing unnecessary biopsies would not only decrease patient discomfort and risks but also have a positive economic impact on the healthcare system. Furthermore, detecting prostate cancer in its early stages can increase survival rates and improve the quality of life for patients.

### Limitations and challenges

Despite promising results, our study is not without limitations and significant challenges. One key challenge was the need to reduce data dimensionality to make our model applicable to a variety of electronic noses rather than being restricted to MOOSY-32. We addressed this challenge by creating a model based on robust curve data obtained from urine samples. However, it is important to note that generalizing our approach to other electronic noses may require additional adjustments and validation in each specific case. Additionally, to bring our methodology into clinical practice, comprehensive physical validation by healthcare professionals is needed, involving a rigorous process that faces regulatory and ethical challenges that must be carefully and diligently addressed.

### Comparison with other prostate cancer detection methods

Our neural network stands out when compared to other prostate cancer detection methods due to its high precision and non-invasive approach. It is crucial to emphasize that our approach is based on using urine samples different from those of the patient being tested. In this context, our primary goal lies in capturing and analyzing sensor responses to specific olfactory patterns. In this regard, we have achieved a significant breakthrough by employing redundancy techniques that substantially enhance the capability of our neural network to detect prostate cancer. Furthermore, parameter reduction has allowed us to work with a 32-dimensional input in the network, contributing to its improved performance. While our model has been specifically developed for data generated by MOOSY-32, we believe that the underlying methodology has the potential to be successfully adapted and applied to other datasets from various electronic noses equipped with metal oxide semiconductor sensors. This advancement paves the way for the implementation of an embedded, low-cost diagnostic system that could be used in outpatient surgery centers and similar clinical settings.

### Future applications

Looking ahead, we consider that our methodology could be adapted to a wide variety of devices, including those equipped with a single sensor or a limited number of sensors. The ability to capture and analyze sensor response curves, precisely extracting 32 key parameters, lays the foundation for the creation of intelligent embedded devices in various fields of medicine and beyond. This technology could drive innovation in the early detection of several diseases, enabling more accessible and effective diagnostic solutions across a broad spectrum of clinical and medical applications.

In Summary, our research represents a significant advancement in non-invasive prostate cancer detection. While we face challenges and limitations, we believe that this methodology has the potential to transform medical practice and improve the lives of prostate cancer patients.

## Conclusion

The neural network that we developed was able to effectively classify instances of prostate cancer with high accuracy. This technique has the potential to decrease the number of biopsies required and make the diagnostic process less invasive. Addition- ally, the training dataset used in this study contained samples from patients across a range of pathological states, making the neural network suitable for classifying prostate cancer in all stages. It is important to note that the neural network is specific to the data obtained from the MOOSY-32 electronic nose, but the methodology used in this study can be applied to datasets from other electronic noses with metal oxide semicon- ductor sensors. This opens up the possibility of implementing a low-cost, embedded diagnostic system for use in outpatient surgery centers.

## Data Availability

The data set used in the study is publicly available at https://github.com/juatafe/e-Nose, and its utilization adhered to the terms and conditions set forth by the data repository.

## References

[1] Tchetgen MBN, Oesterling JE (1997). The effect of prostatitis, urinary retention, ejaculation, and ambulation on the serum prostate-specific antigen concentration. Urol Clin North Am.

[2] Bostanci Y, Kazzazi A, Momtahen S, Laze J, Djavan B (2013). Correlation between benign prostatic hyperplasia and inflammation. Curr Opin Urol.

[3] Ankerst DP. et al. Serial Percent-Free PSA in Combination with PSA for Population-Based Early Detection of Prostate Cancer. J Urol. 2016. 10.1016/j.juro.2016.03.011 PMC4969186 26979652

[4] Jia XM, Meng QH, Jing YQ, Qi PF, Zeng M, Ma SG. A new method combining KECA-LDA with ELM for classification of Chinese liquors using electronic nose. IEEE Sens J 2016;99.

[5] Jing Y, Meng Q, Qi P, Cao M, Zeng M, Ma S (2016). A bioinspired neural net- work for data processing in an electronic nose. IEEE Trans Neural Netw Learn Syst.

[6] Sreelatha M, Nasira GM, Thangamani P. Pattern recognition for toxic gases based on electronic nose using artificial neural networks. In: 2016 Int. Conf Comput Sustain Glob Dev 2016:3075–3079.

[7] Tian F (2016). Suppression of strong background interference on e-nose sensors in an open country environment. Sensors (Switzerland).

[8] Chakravarthy ASN. Electronic noses: forestalling fire disasters. 2015.

[9] Wilson AD, Baietto M (2011). Advances in electronic-nose technologies developed for biomedical applications. Sensors.

[10] Win DT. The electronic nose – a big part of our future. 2005;9(1):1–8.

[11] Roine A (2014). Detection of prostate cancer by an electronic nose: a proof of principle study. J Urol.

[12] D'Orazio M (2020). Deciphering cancer cell behavior from motility and shape features: Peer prediction and dynamic selection to support cancer diagnosis and therapy. Front Oncol.

[13] D’Orazio M (2022). Machine learning phenomics (MLP) combining deep learning with time-lapse-microscopy for monitoring colorectal adenocarcinoma cells gene expression and drug-response. Sci Rep.

[14] Mencattini A (2023). Deep-Manager: a versatile tool for optimal feature selection in live-cell imaging analysis. Commun Biol.

[15] Cupane M, Sebastia JP (2015). Application of MOOSY32 eNose to assess the effects of some post harvest treatments on the quality of ‘Salustiana’ orange juice. J Biosens Bioelectron..

[16] TGS 2611-E00 - for the detection of Methane no. TGS 2611-E00.

[17] “TGS 2611-C00 - for the detection of Methane,” no. TGS 2611-C00.

[18] “TGS 2610-C00 and TGS 2610-D00 - for the detection of LP Gas,” no. TGS 2610.

[19] Fígaro USA INC, “TGS 2620 for the detection of Solvent Vapors,” Prod. Inf., 2014.

[20] Abadi M et al. TensorFlow: Large-scale machine learning on heteroge- neous systems. 2015.

[21] Krizhevsky A, Sutskever I, Hinton GE. AlexNet. Adv Neural Inf Process Syst. 2012.

